# Green Tea and Java Pepper Mixture Prevents Obesity by Increasing Energy Expenditure and Modulating Hepatic AMPK/MicroRNA-34a/370 Pathway in High-Fat Diet-Fed Rats

**DOI:** 10.3390/antiox12051053

**Published:** 2023-05-05

**Authors:** Jibin Kim, Dahye Han, Mak-Soon Lee, Jumi Lee, In-Hwan Kim, Yangha Kim

**Affiliations:** 1 Department of Nutritional Science and Food Management, Ewha Womans University, Seoul 03760, Republic of Korea; jbin1004@ewhain.net (J.K.); had02167@ewhain.net (D.H.); troph@ewha.ac.kr (M.-S.L.); epiecess95@ewhain.net (J.L.); 2 Graduate Program in System Health Science and Engineering, Department of Nutritional Science and Food Management, Ewha Womans University, Seoul 03760, Republic of Korea; 3 Department of Integrated Biomedical and Life Science, Graduate School, Korea University, Seoul 02841, Republic of Korea; k610in@korea.ac.kr

**Keywords:** green tea, java pepper, obesity, microRNA, AMPK, energy expenditure

## Abstract

This study was performed to evaluate the anti-obesity effects of green tea and java pepper mixture (GJ) on energy expenditure and understand the regulatory mechanisms of AMP-activated protein kinase (AMPK), microRNA (miR)-34a, and miR-370 pathways in the liver. Sprague–Dawley rats were divided into four groups depending on the following diets given for 14 weeks: normal chow diet (NR), 45% high-fat diet (HF), HF + 0.1% GJ (GJL), and HF + 0.2% GJ (GJH). The results revealed that GJ supplementation reduced body weight and hepatic fat accumulation, improved serum lipids, and increased energy expenditure. In the GJ-supplemented groups, the mRNA levels of genes related to fatty acid syntheses, such as a cluster of differentiation 36 (CD36), sterol regulatory element binding protein-1c (SREBP-1c), fatty acid synthase (FAS), and stearoyl-CoA desaturase 1 (SCD1) were downregulated, and mRNA levels of peroxisome proliferator-activated receptor alpha (PPARα), carnitine/palmitoyl-transferase 1 (CPT1), and uncoupling protein 2 (UCP2), which participate in fatty acid oxidation, were upregulated in the liver. GJ increased the AMPK activity and decreased the miR-34a and miR-370 expression. Therefore, GJ prevented obesity by increasing energy expenditure and regulating hepatic fatty acid synthesis and oxidation, suggesting that GJ is partially regulated through AMPK, miR-34a, and miR-370 pathways in the liver.

## 1. Introduction

Obesity can be complexly involved in various causes, including biological factors such as genetics, physiology, and metabolism, and psychosocial factors such as mental, behavioral, social, and cultural factors [1,2]. Moreover, obesity can result from an imbalance between energy intake and energy expenditure; it is associated with metabolic diseases such as type 2 diabetes, non-alcoholic fatty liver disease, and hyperlipidemia [3]. The liver plays an important role in the regulation of energy expenditure and lipid metabolism [4]. Chronically imbalanced energy leads to fat accumulation in the liver, thereby impairing metabolic control functions [5]. Recently, studies have comprehensively explored obesity therapeutics that aim to increase energy expenditure by regulating fatty acid β-oxidation in the liver [5,6]. Therefore, the mechanisms of energy expenditure and hepatic fatty acid oxidation during the development of obesity should be elucidated to prevent and treat obesity.

AMP-activated protein kinase (AMPK) regulates energy homeostasis and fatty acid oxidation in the liver [7]. Obesity caused by energy imbalance not only exacerbates metabolic disorders but also reduces AMPK activity [8]. The increased activity of AMPK reduces hepatic triglyceride (TG) and downregulates lipogenic genes such as sterol regulatory element binding protein-1c (SREBP-1c), fatty acid synthase (FAS), and stearoyl-CoA desaturase 1 (SCD1) [9]. AMPK activation also stimulates hepatic fatty acid β-oxidation by upregulating peroxisome proliferator-activated receptor alpha (PPARα), carnitine/palmitoyl-transferase 1 (CPT1), and uncoupling protein 2 (UCP2) [7,10].

MicroRNA (miR) is a small non-coding RNA consisting of 18–22 nucleotide molecules that play a role as a post-transcriptional regulator in regulating gene expression [11]. miRs have been widely recognized as therapeutic targets because they regulate metabolism and affect various metabolic diseases [12]. miR-34a is highly expressed in the fatty liver of mice and directly targets the transcription factor PPARα related to fatty acid oxidation [12]. Furthermore, miR-370 regulates the CPT1 expression by targeting the 3ʹ-untranslated region of CPT1 that affects lipid metabolism [11]. Thus, miR-34a and miR-370 may be used as target biomarkers to regulate fatty acid oxidation.

Green tea (*Camellia sinensis*) is mainly consumed worldwide as a tea beverage. It contains catechin, a physiologically active polyphenol, which representatively includes epigallocatechin (EGC), epicatechin (EC), epigallocatechin-3-gallate (EGCG), and epicatechin-3-gallate (ECG). Green tea catechins have health-promoting properties such as antioxidant and anti-obesity effects [13]. EGCG, in particular, which has the highest antioxidant activity in green tea, helps reduce weight and prevents fatty liver by downregulating lipogenesis and upregulating lipolysis in mice fed with a high-fat (HF) diet [14,15].

Java pepper (*Piper retrofractum*), a plant belonging to Piperaceae, is widely cultivated in Southeast Asia; it is commonly dried and used as a spice. Piperine, a major component of java pepper, improves dyslipidemia and has anti-obesity and antioxidative effects [16]. In particular, piperine enhances the low bioavailability of EGCG [17]. EGCG reduces colitis by enhancing its anti-inflammatory effect when it is administered with piperine [18]. We reported the anti-colitis effect of green tea and java pepper mixture (GJ) by modulating colonic miR-21 in dextran sulfate sodium-induced colitis [19]. In the present study, we investigated the anti-obesity effect of GJ in HF diet-fed rats. We also determined whether GJ supplementation promoted energy expenditure and affected hepatic lipid metabolism by regulating AMPK and miR-34a/370 pathways.

## 2. Materials and Methods

### 2.1. Preparation of Green Tea and Java Pepper Mixture (GJ)

GJ was a mixture of green tea and java pepper extracts supplied by Newtree (Seoul, Republic of Korea), as described in our previous study [19]. Green tea extract was obtained from Naturex (Avignon, France). Java pepper was extracted with 70% ethanol at 60 °C for 8 h and mixed at a ratio of 99:1 (*w*/*w*). The mixtures were sterilized at 90–95 °C for 15–30 min and spray-dried, then the catechins and piperine contained in this powder were determined. The contents of catechins and piperine in green tea and java pepper mixture (GJ) were analyzed by high-performance liquid chromatography (HPLC) analysis using a Nanospace SI-2 HPLC system (Shiseido Co., Tokyo, Japan) [19]. The analytical conditions were as follows: column: Capcell Pak C18 UG 120 column (4.6 mm × 250 mm, 5 µm; Shiseido, Tokyo, Japan); flow rate: 1 mL/min; column temperature: 35 °C; volume of injection: 10 μL; detector wavelength: 343 nm. The epigallocatechin-3-gallate (EGCG), epicatechin-3-gallate (ECG), epigallocatechin (EGC), and epicatechin (EC) contents of GJ were 522.04 ± 9.84, 111.22 ± 2.06, 105.67 ± 2.51, and 53.06 ± 1.09 mg/g, respectively. The piperine content of GJ was 2.05 ± 0.13 mg/g. Data are expressed as mean ± standard error of six replicates [19].

### 2.2. Animals and Diets

Animal experiments were performed according to those previously described [20], and experimental procedures were approved by the Institutional Animal Care and Use Committee (IACUC) of Ewha Womans University (IACUC No. 20-015, accessed on 23 March 2020). Three-week-old Sprague–Dawley male rats (Doo Yeol Biotech Co., Seoul, Republic of Korea) were individually housed in cages under the following conditions: temperature (22 ± 2 °C), humidity (55% ± 5%), and 12 h/12 h light/dark cycle. After being acclimatized to a normal chow diet (2018S Teklad rodent diet, Envigo, Indianapolis, IN, USA) with free access to water for 1 week, the rats were randomly divided into four groups (*n* = 7/group). Experimental diets were given twice a week as follows and allowed ad libitum: normal chow diet (NR), 45% HF diet, HF containing 0.1% GJ (GJL), and HF containing 0.2% GJ (GJH). Table S1 shows the compositions of the experimental diets in each group. The body weight and food intake of the rats were measured weekly. After the 14-week experimental period, the rats were anesthetized with a mixture of Zoletil 50 (Virbac Laboratories, Carros, France) and Rompun (Bayer Korea, Seoul, Republic of Korea) at a ratio of 5:2 (*v*/*v*). Whole blood collected via cardiac puncture was allowed to coagulate at room temperature for 1 h. Serum was separated from the supernatant by centrifugation at 1500× *g* (20 min, 4 °C) and stored at −40 °C until analysis. The excised liver and white adipose tissue (WAT) samples were immediately frozen in liquid nitrogen and stored at −70 °C until analysis.

### 2.3. Measurement of Serum Metabolic Parameters

The serum concentrations of TG, total cholesterol (TC), and high-density lipoprotein cholesterol (HDL-C) were measured using commercially available kits (Asan Pharmaceutical Co., Seoul, Republic of Korea), as previously described [20]. The level of low-density lipoprotein cholesterol (LDL-C) was calculated using the Friedewald formula: LDL-C = TC–HDL-C–(TG/5) [21]. The serum level of non-esterified fatty acid (NEFA) was measured using another commercial kit (Wako Pure Chemical, Osaka, Japan). The serum levels of aspartate aminotransferase (AST) and alanine transaminase (ALT) were determined on the basis of the enzymatic colorimetric method by using commercial kits (Embiel, Gunpo, Republic of Korea).

### 2.4. Measurement of Energy Expenditure

After 12 weeks, each rat was housed in an individual metabolic cage with free access to food and water. O_2_ and CO_2_ analyzers were calibrated with purified gas before measurement [22]. Oxygen consumption (VO_2_) and carbon dioxide production (VCO_2_) were determined via indirect calorimetry (Oxylet, Panlab, Barcelona, Spain). VO_2_ and VCO_2_ data were recorded for 24 h at 3 min intervals using a computer-assisted data acquisition program (Chart 5.2; AD Instrument, Bella Vista, NSW, Australia). Energy expenditure (EE) was obtained according to the following formula:

(1) EE kcal/day/kg^0.75=3.815×VO2+1.232×VCO2×1.44.

### 2.5. Hematoxylin and Eosin (H&E) Staining

Epididymal WAT (eWAT) and liver tissues were fixed in 10% formalin solution overnight. The fixed tissues were embedded in a paraffin block, sectioned, and stained with H&E. The stained sections were observed under a microscope (200× magnification; Olympus, Tokyo, Japan). H&E staining images were analyzed using Image J 1.51k software (produced by Wayne Rasband, United States National Institutes of Health, Bethesda, MD, USA) to measure the size of the adipocyte area in eWAT.

### 2.6. Hepatic Lipid Analysis

Hepatic lipids were extracted by modifying the Bligh and Dyer method [23]. In this procedure, 0.5 g of liver tissue was homogenized with 1.5 mL of 0.9% saline, and 7.5 mL of chloroform and methanol were added to the homogenized sample at a ratio of 1:2 (*v*/*v*). After 10 min of vortexing, 2.5 mL of chloroform was added and centrifuged at 3000 rpm for 20 min to collect the lower aqueous layer by using a Pasteur pipette. The extracted lipids were filtered into a beaker by using a No. 6 filter paper (Whatman International Ltd., Maidstone, UK), dried, and weighed. The hepatic lipids were dissolved in 5 mL of n-hexane: isopropanol solution at 3:2 (*v*/*v*) and stored at −40 °C until analysis. Hepatic TG and TC levels were measured using commercial kits as described above.

### 2.7. Real-Time Quantitative Polymerase Chain Reaction (RT-qPCR)

For RT-qPCR, total RNA was extracted from the eWAT and liver by using TRIzol reagent (GeneAll Biotechnology, Seoul, Republic of Korea) [20]. cDNA was synthesized with M-MLV reverse transcriptase (Bioneer, Daejeon, Republic of Korea) by using an mRNA and cDNA synthesis kit with poly (A) polymerase tailing (ABM Inc., Richmond, BC, Canada) for miR. Next, RT-qPCR was performed with a Rotor-Gene Q thermocycler (Qiagen, Hilden, Germany) by adding Greenstar qPCR Master Mix (Bioneer). Table S2 lists the primer sequences designed by Primer3 [24], and mRNA expression was normalized using β-actin as a reference control. The specific primers of miR-34a, miR-370, and RNU6 were purchased from ABM Inc., and the expression levels of miRs were normalized using the expression of RNU6 snRNA as a control. The mRNA and miR expression levels were calculated via the 2^−∆∆Ct^ method for relative quantification [25]. They were then expressed as fold differences compared with those of the HF group.

### 2.8. AMPK Activity

The AMPK activity was determined by assaying the total protein lysates from the liver in an AMPK assay kit (Cyclex, Nagano, Japan), as previously described [26]. The AMPK activity was normalized to the protein concentration determined with a bicinchoninic acid (BCA) protein assay kit (Thermo Scientific, Rockford, IL, USA) and expressed as fold changes in the HF group.

### 2.9. Statistical Analysis

Statistical analysis was performed using SPSS version 25 (SPSS Inc., Chicago, IL, USA). Data were expressed as mean ± standard error of the mean (SEM) for seven animals in each group. Significant differences in the three groups (HF, GJL, and GJH groups) were analyzed via one-way ANOVA followed by Tukey’s multiple comparison tests. Data with *p* < 0.05 were considered significant. Significant differences between the NR and HF groups were determined by a one-tailed Student’s *t*-test. Data with * *p* < 0.05, ** *p* < 0.01, and *** *p* < 0.001 were considered significant.

## 3. Results

### 3.1. Effects of GJ on Body Weight Gain and Energy Intake

**Figure 1:**
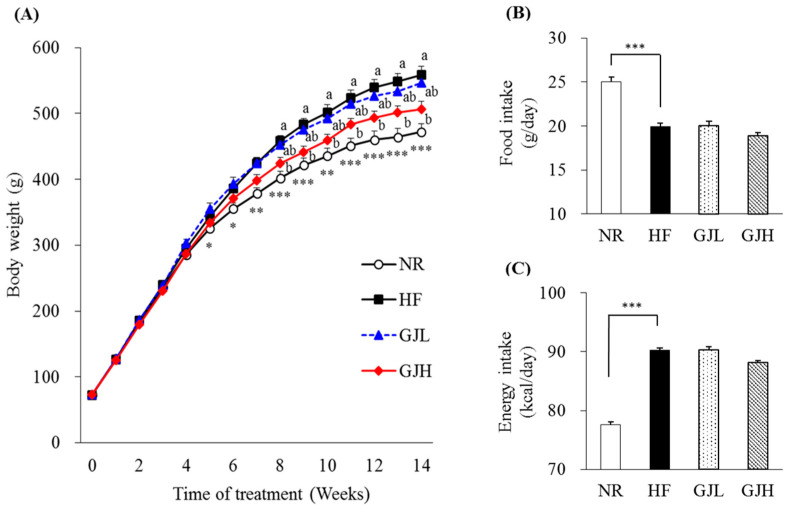
Effects of GJ on body weight, food intake, and energy intake: changes in body weight during the experimental period in mice fed NR (open circle), HF (black square), GJL (blue triangle) and GJH (red diamond) (**A**), food intake (**B**), and energy intake (**C**). Data are expressed as mean ± SEM (*n* = 7). Different letters indicate significant differences among the three groups (HF, GPL, and GPH) at *p* < 0.05. Significant differences between the NR and HF groups are indicated: * *p* < 0.05, ** *p* < 0.01, *** *p* < 0.001. NR—normal chow diet; HF—45% high-fat diet; GJL—HF with 0.1% GJ; GJH—HF with 0.2% GJ.

After 14 weeks of the experiment, the final body weight and weight gain in the GJH group were reduced by 9.5% and 11.4% compared with those of the HF group, respectively (*p* < 0.05; Table 1). From the eighth week, the weight of the GJH group reduced by 7.5% compared with that of the HF group (*p* < 0.05; Figure 1A). The liver weight of the GJH group also decreased by 12.4% compared with that of the HF group (*p* < 0.05; Table 1). Food and energy intake did not differ among the experimental groups (HF, GJL, and GJH; Figure 1B,C).

### 3.2. Effects of GJ on Serum Metabolic Parameters

Serum metabolite profiles are shown in Table 2. The serum TG levels of the GJL and GJH groups were 32.8% and 33.4% lower than those of the HF group, respectively (*p* < 0.05). The serum TC and LDL-C levels of the GJH groups were 25.3% and 68.7% lower than those of the HF group, respectively (*p* < 0.05). The NEFA level of the GJH group was reduced by 19.1% compared with that of the HF group (*p* < 0.05). However, serum AST and ALT levels, which are hepatotoxicity indicators, did not significantly change among the groups.

### 3.3. Effect of GJ on Energy Expenditure

**Figure 2:**
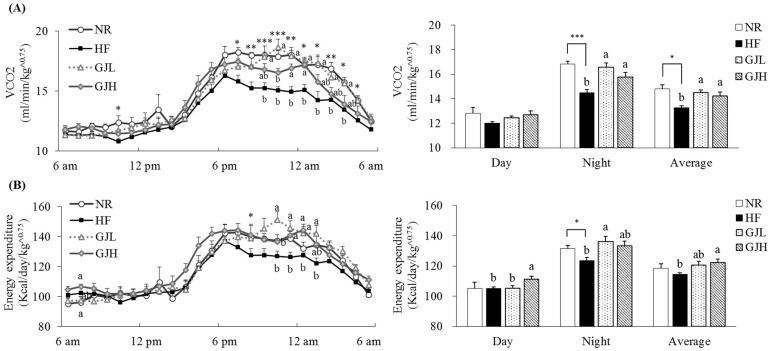
Effects of GJ on VCO_2_ (**A**) and energy expenditure (**B**). Data are expressed as mean ± SEM (*n* = 7). Different letters indicate significant differences among the three groups (HF, GJL, and GJH) at *p* < 0.05. Significant differences between the NR and HF groups are indicated: * *p* < 0.05, ** *p* < 0.01, *** *p* < 0.001. NR—normal chow diet; HF—45% high-fat diet; GJL—HF with 0.1% GJ; GJH—HF with 0.2% GJ; VCO_2_—carbon dioxide production.

We evaluated the effects of GJ on energy expenditure (Figure 2). VCO_2_ observed in seven rats per group was 9.4% and 7.4% higher in the GJL and GJH groups than those of the HF group, respectively (*p* < 0.05; Figure 2A). The energy expenditure in the GJH group was 7.0% higher than that of the HF group. (*p* < 0.05; Figure 2B).

### 3.4. Effects of GJ on Fat Deposition and Adipogenic Gene Expression in WAT

**Figure 3:**
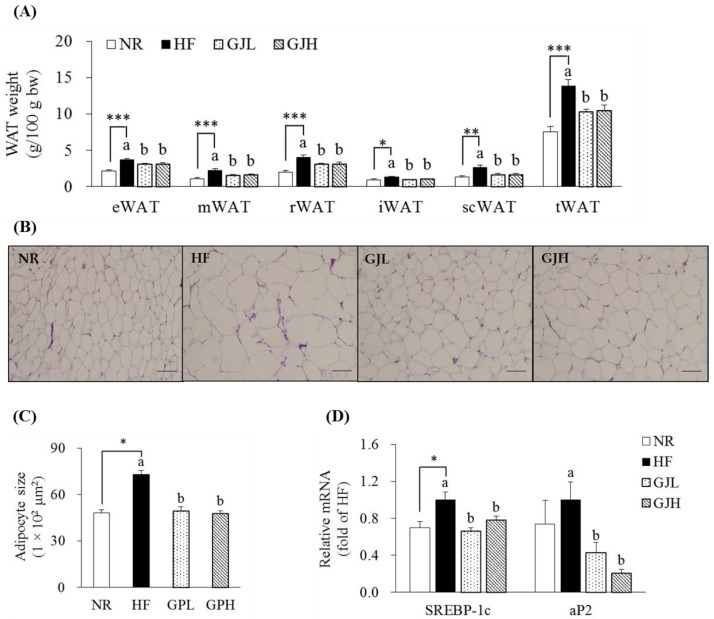
Effects of GJ on fat deposition (**A**–**C**) and adipogenic gene expression (**D**) in eWAT. (**B**) Representative images of the H&E-stained eWAT section (scale bar = 100 μm; magnification of 200×). (**C**) Adipocyte size (area per adipocyte, μm^2^). Data are expressed as mean ± SEM (*n* = 7). Different letters indicate significant differences among the three groups (HF, GJL, and GJH groups) at *p* < 0.05. Significant differences between the NR and HF groups are indicated: * *p* < 0.05, ** *p* < 0.01, *** *p* < 0.001. NR—normal chow diet; HF—45% high-fat diet; GJL—HF with 0.1% GJ; GJH—HF with 0.2% GJ; eWAT—epididymal white adipose tissue; mWAT—mesenteric white adipose tissue; rWAT—retroperitoneal white adipose tissue; iWAT—inguinal white adipose tissue; scWAT—subcutaneous white adipose tissue; tWAT—total white adipose tissue.

The masses of epididymal WAT (eWAT), mesenteric WAT (mWAT), retroperitoneal WAT (rWAT), inguinal WAT (iWAT), subcutaneous WAT (scWAT), and total WAT (tWAT) in the HF group increased compared with those in the NR group (*p* < 0.05; Figure 3A). However, all WAT masses decreased in the GJL and GJH groups compared with those in the HF group (*p* < 0.05). In Figure 3B,C, the adipocyte sizes of the eWAT enlarged by the HF diet were reduced by 32.7% and 35.0% in the GJL and GJH groups, respectively (*p* < 0.05). Considering that adipocyte hypertrophy in eWAT was inhibited by GJ, we measured the mRNA levels of adipogenic genes in eWAT (Figure 3D). The SREBP-1c expression levels in the GJL and GJH groups were downregulated by 34.1% and 22.2% compared with those in the HF group, respectively (*p* < 0.05). The mRNA levels of adipocyte protein 2 (aP2) in the GJL and GJH groups were downregulated by 57.5% and 79.4%, respectively (*p* < 0.05).

### 3.5. Effects of GJ on Lipid Accumulation and Gene Expression Related to Lipid Metabolism in the Liver

**Figure 4:**
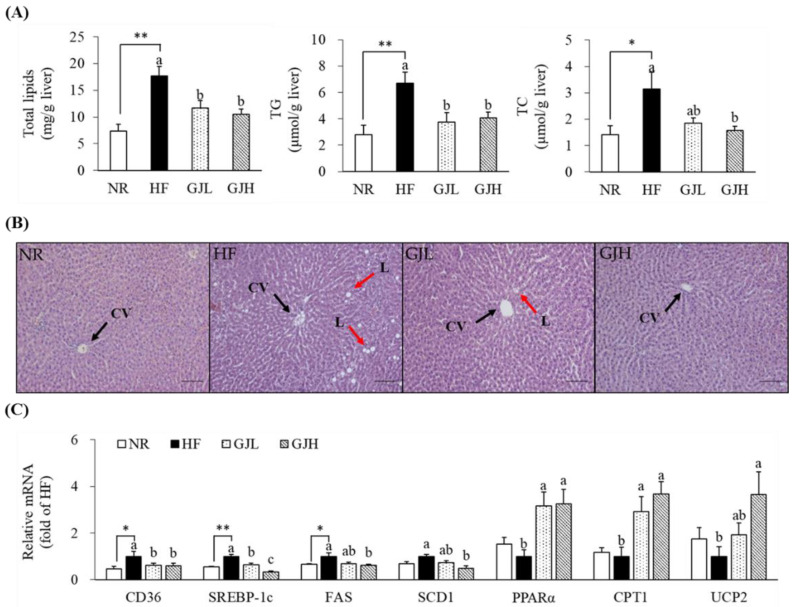
Effects of GJ on lipid accumulation (**A**,**B**) and gene expression related to lipid metabolism (**C**) in the liver. (**B**) Representative images of the H&E-stained liver section (scale bar = 100 μm; magnification of 200×); liver showing CV (black arrow) and L (red arrow). Data are expressed as mean ± SEM (*n* = 7). Different letters indicate significant differences among the three groups (HF, GJL, and GJH) at *p* < 0.05. Significant differences between the NR and HF groups are indicated: * *p* < 0.05, ** *p* < 0.01. NR—normal chow diet; HF—45% high-fat diet; GJL—HF with 0.1% GJ; GJH—HF with 0.2% GJ; CV—central vein; L—lipid droplet.

To investigate the effect of GJ on hepatic lipid accumulation, we measured hepatic lipid profiles and observed H&E staining (Figure 4A,B). The total lipid levels decreased in the GJL and GJH groups by 33.6% and 40.6% compared with those in the HF group, respectively (*p* < 0.05). The hepatic TG concentrations increased by the HF diet were decreased in the GJL and GJH groups by 43.9% and 39.2% compared with those of the HF group, respectively (*p* < 0.05). The liver TC concentration in the GJH group was reduced by 50.4% compared with that in the HF group (*p* < 0.05). GP-supplemented groups tended to reduce the number and size of HF diet-induced hepatic lipid droplets. The mRNA levels related to lipid metabolism in the liver were analyzed by RT-qPCR (Figure 4C). The gene expression levels of CD36, a transporter of free fatty acid uptake, were downregulated in the GPL and GPH groups by 38.3% and 41.1% compared with those in the HF group, respectively (*p* < 0.05). The mRNA levels of SREBP-1c, FAS, and SCD1, which are lipogenesis-related genes, were downregulated in the GJH group by 66.6%, 39.2%, and 52.1% compared with those in the HF group, respectively (*p* < 0.05). The mRNA levels of genes involved in fatty acid oxidation, such as PPARα, CPT1, and UCP2, were upregulated by 3.25-, 3.67-, and 3.66-fold in the GJH group compared with those in the HF group, respectively (*p* < 0.05).

### 3.6. Effects of GJ on AMPK Activity and miR-34a/370 Expression

**Figure 5:**
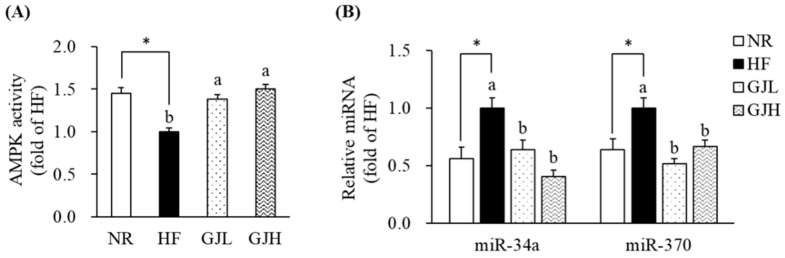
Effects of GJ on AMPK activity (**A**) and miR-34a/370 expression (**B**). Data are expressed as mean ± SEM (*n* = 7). Different letters indicate significant differences among the three groups (HF, GJL, and GJH) at *p* < 0.05. Significant differences between the NR and HF groups are indicated: * *p* < 0.05. NR—normal chow diet; HF—45% high-fat diet; GJL—HF with 0.1% GJ; GJH—HF with 0.2% GJ.

To investigate the mechanism by which GJ regulates fatty acid oxidation, we further analyzed the AMPK activity and the miR-34a and miR-370 expression in the liver. The AMPK activities reduced by the HF diet were increased in the GJL and GJH groups by 1.38- and 1.50-fold compared with those in the HF group, respectively (*p* < 0.05; Figure 5A). In Figure 5B, the miR-34a and miR-370 expression levels were higher in the HF group than in the NR group (*p* < 0.05; Figure 5B). However, the miR-34a levels were lower by 35.7% and 59.6% in the GJL and GJH groups than in the HF group, respectively (*p* < 0.05). The miR-370 levels were also lower by 48.1% and 33.2% in the GJL and GJH groups than in the HF group, respectively (*p* < 0.05).

## 4. Discussion

Excessive energy intake disrupts the metabolic regulation of the developmental process of obesity and leads to obesity; thus, increasing energy expenditure is effective in preventing obesity [6,27]. This study was performed to investigate the effects of GJ on energy expenditure and the regulation of AMPK and miR pathways in HF diet-fed obese rats. Among the bioactive compounds contained in green tea, the main antioxidant agents are catechins [13]. The number of total catechins, including EGCG, ECG, EGC, and EC, identified in GJ was 792.00 ± 15.43 g/kg, and the amount of piperine was 2.05 ± 0.13 g/kg. Among the bioactive components of GJ, EGCG had the highest content at 522.04 ± 9.84 g/kg. GJ supplementation reduced the body weight and WAT weight without altering food intake and energy intake. GJ improved serum and liver lipid profiles and increased energy expenditure. Previous studies reported that green tea extract decreases body weight and improves serum and hepatic lipid profiles in high-energy or HF diet-induced obesity [28,29,30]. In addition, EGCG, which is abundant in green tea, increases energy expenditure while reducing body weight and serum lipid levels in mice fed with an HF diet [14,31]. Recent studies have reported that piperine reduces HF diet-induced weight gain and improves serum and liver lipid profiles [32,33]. Our results suggested that GJ containing EGCG and piperine improved serum and hepatic lipid levels and inhibited weight gain by increasing energy expenditure.

Energy imbalance, a state in which energy intake is higher than consumption, causes fat accumulation in adipose tissues [27]. SREBP-1c and aP2 participate in adipogenesis toward fat accumulation [34]. SREBP-1c is a transcription factor of adipogenic gene expression [35]. aP2 is a fatty acid transporter protein upregulated during adipocyte differentiation [36]. GJ downregulated the mRNA levels of SREBP-1c and aP2 in WAT. In a previous study, green tea catechins downregulate the mRNA levels of SREBP-1c and aP2 in WAT [37]. Du et al. [32] showed that piperine downregulates the mRNA levels of SREBP-1c in the WAT of HF diet-induced obese mice. Our results indicated that GJ might regulate adipogenic genes to suppress fat accumulation in WAT.

The liver is the major organ involved in lipid metabolism and is responsible for fatty acid uptake, synthesis, and oxidation [38]. The AMPK activity in the liver affects lipid metabolism by decreasing fatty acid synthesis and increasing fatty acid oxidation [7,39]. AMPK maintains cellular fatty acid homeostasis by regulating CD36, which uptakes circulating fatty acids in the blood into the liver [40]. It inhibits SREBP-1c, which activates the transcription of FAS and SCD1 genes involved in fatty acid and TG synthesis in the liver [9,41]. It also increases fatty acid oxidation ability by regulating PPARα and CPT1 [42,43]. PPARα activates the regulatory enzyme CPT1, which transports cytosolic fatty acids to the mitochondria and increases UCP2 to induce fatty acid oxidation [44,45]. In particular, the mRNA transcription levels of CD36, SREBP-1c, FAS, SCD1, PPARα, CPT1, and UCP2 genes in the liver were consistent with their protein expression levels [46,47,48,49]. Green tea extract activates AMPK and reduces the expression of SREBP-1c and FAS proteins in the liver of HF diet-fed mice [50]. In addition, green tea and its catechins downregulate the expression of CD36 and SCD1, upregulate the expression of PPARα, CPT1, and UCP2, and activate AMPK in the liver [51,52]. EGCG alleviates hepatic fat deposition by enhancing AMPK activity and downregulating SREBP-1c-mediated lipid biosynthesis in the liver [53]. Kim et al. [54] reported that EGCG, ECG, EGC, and EC inhibit lipid accumulation in mature adipocytes, and in particular, EGC exerts an anti-obesity effect by inducing UCP1 expression. Piperine decreases the mRNA levels of hepatic SREBP-1c, FAS, and CD36 but increases CPT1 expression and AMPK activation in HF diet-induced obesity [33,55]. Consistent with the above results, our results showed that GJ increased the activation of AMPK, downregulated the expression of CD36, SREBP-1c, FAS, and SCD1, and upregulated the expression of PPARα, CPT1, and UCP2. On the basis of these results, we proposed that the increased energy expenditure by GJ was related to the stimulation of AMPK activity and fatty acid oxidation in the liver.

miRs have been extensively studied because they affect the expression of genes involved in various metabolisms [56]. In particular, miR-34a and miR-370 inhibit fatty acid oxidation by directly targeting PPARα and CPT1, respectively [11,12]. Specifically, silencing miR-34a increases the AMPK phosphorylation pathway in the liver of HF diet-fed mice [12]. Torres et al. [52] demonstrated that green tea upregulates its direct target PPARα by downregulating miR-34a in HF diet-fed mice. The present study is the first to demonstrate that GJ inhibited the expression of miR-34a and miR-370 in the liver. These results implied that GJ might mediate fatty acid oxidation partly by regulating the miR-34a and miR-370 pathways in the liver.

This study has some limitations. At this stage of the study, male rats were selected to eliminate possible variations in food intake during the female oestrus cycle, which would be difficult to control [57]. Since the scope of this study is limited to the effect of GJ on increasing energy expenditure through the hepatic regulatory mechanism, additional research on brown adipose tissue, which is known to be an energy expenditure target, will be needed. Furthermore, based on animal experimental results, future translational research on its safety and clinical application should be performed.

## 5. Conclusions

**Figure 6:**
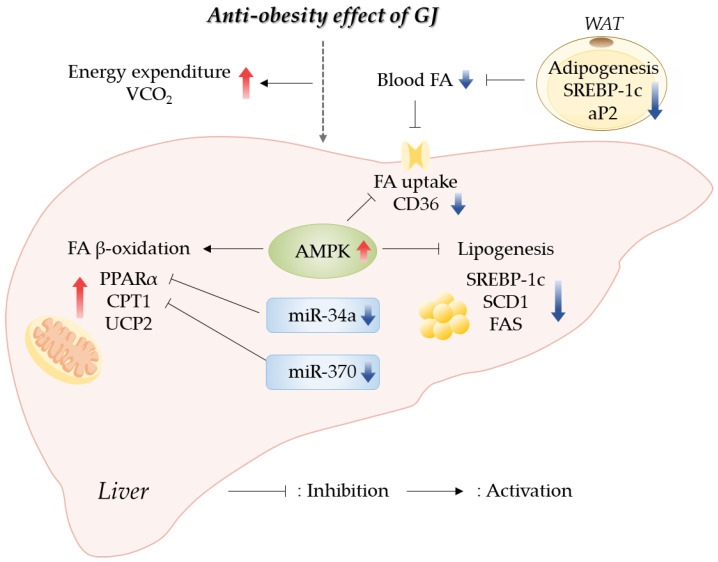
Schematic of GJ-mediated regulatory mechanisms preventing obesity in rats fed with a high-fat diet; GJ—green tea and java pepper mixture; AMPK—AMP-activated protein kinase; aP2—adipocyte protein 2; CD36—cluster of differentiation 36; CPT1—carnitine/palmitoyl-transferase 1; FA—fatty acid; FAS—fatty acid synthase; miR—microRNA; PPARα—peroxisome proliferator-activated receptor alpha; SCD1—stearoyl-CoA desaturase 1; SREBP-1c—sterol regulatory element binding protein-1c; UCP2—uncoupling protein 2; VCO_2_—carbon dioxide production; WAT—white adipose tissue.

Our results indicated that GJ prevented obesity through an increase in energy expenditure by promoting hepatic fatty acid oxidation and a reduction in lipid accumulation in the WAT and liver. GJ supplementation upregulated the expression of genes related to fatty acid oxidation and downregulated the expression of genes related to fatty acid synthesis in the liver. Moreover, we reported for the first time that the anti-obesity effect of GJ might be partially involved in AMPK and miR-34a/370 regulatory pathways (Figure 6). Therefore, GJ could be useful as a candidate material for ameliorating obesity-related lipid and energy metabolism.

## Figures and Tables

**Table 1:** Effects of GJ on weight gain and liver weight.

Variables	NR	HF	GJL	GJH
Initial weight (g)	72.64 ± 2.04	72.58 ± 2.03	72.79 ± 1.88	72.66 ± 2.01
Final weight (g)	472.25 ± 12.03 ***	558.95 ± 12.93 ^a^	546.21 ± 9.55 ^ab^	506.12 ± 11.91 ^b^
Weight gain (g)	400.39 ± 12.27 ***	487.10 ± 13.09 ^a^	472.22 ± 8.36 ^a^	431.75 ± 11.55 ^b^
Liver weight (g)	13.44 ± 0.40	14.49 ± 0.38 ^a^	14.18 ± 0.47 ^a^	12.69 ± 0.50 ^b^

Data are expressed as mean ± SEM (*n* = 7). ^a,b^ Different letters within a row indicate significant differences among the three groups (HF, GJL, and GJH) at *p* < 0.05. Significant differences between the NR and HF groups within a row are indicated *** *p* < 0.001. NR—normal chow diet; HF—45% high-fat diet; GJL—HF with 0.1% GJ; GJH—HF with 0.2% GJ.

**Table 2:** Effects of GJ on serum metabolic parameters.

Variables	NR	HF	GJL	GJH
TG (mmol/L)	0.92 ± 0.10	1.16 ± 0.14 ^a^	0.78 ± 0.04 ^b^	0.77 ± 0.08 ^b^
TC (mmol/L)	2.79 ± 0.07	2.63 ± 0.14 ^a^	2.28 ± 0.15 ^ab^	1.97 ± 0.05 ^b^
HDL-C (mmol/L)	1.71 ± 0.11 *	1.34 ± 0.07	1.35 ± 0.10	1.37 ± 0.03
LDL-C (mmol/L)	0.66 ± 0.12	0.76 ± 0.16 ^a^	0.58 ± 0.13 ^ab^	0.24 ± 0.05 ^b^
NEFA (mEq/L)	0.69 ± 0.03	0.69 ± 0.03 ^a^	0.60 ± 0.03 ^ab^	0.56 ± 0.03 ^b^
AST (IU/L)	72.31 ± 4.64	66.20 ± 5.55	59.55 ± 3.64	62.63 ± 3.00
ALT (IU/L)	12.61 ± 1.46	10.63 ± 1.03	9.48 ± 0.49	9.91 ± 0.65

Data are expressed as mean ± SEM (*n* = 7). ^a,b^ Different letters within a row indicate significant differences among the three groups (HF, GJL, and GJH) at *p* < 0.05. Significant differences between the NR and HF groups within a row are indicated * *p* < 0.05. NR—normal chow diet; HF—45% high-fat diet; GJL—HF with 0.1% GJ; GJH—HF with 0.2% GJ. TG—triglyceride; TC—total cholesterol; LDL-C—LDL-cholesterol; HDL-C—HDL-cholesterol; NEFA—non-esterified fatty acids; AST—aspartate aminotransferase; ALT—alanine transaminase.

## Data Availability

All data related to this research are presented in the manuscript.

## Supplementary Materials

The following supporting information can be downloaded at: https://www.mdpi.com/article/10.3390/antiox12051053/s1, Table S1. Compositions of experimental diets; Table S2. Primers used for real-time quantitative reverse transcription PCR.
