# Supplementary material for: Green Tea and Java Pepper Mixture Prevents Obesity by Increasing Energy Expenditure and Modulating Hepatic AMPK/MicroRNA-34a/370 Pathway in High-Fat Diet-Fed Rats
Source: Antioxidants (Basel). 2023 May 5;12(5):1053. doi: 10.3390/antiox12051053 (PMC10215510; doi:10.3390/antiox12051053)
Supplement: Supplementary file 1 [file antioxidants-12-01053-s001.zip › antioxidants-2312531-supplementary.pdf]

Supplementary Data

Table S1. The compositions of experimental diets

(g / kg diet)

**Component**	**HF**	**GJL**	**GJH**
Casein	170.73	170.73	170.73
Sucrose	121.95	121.95	121.95
Dextrose	154.00	154.00	154.00
Corn starch	201.71	200.71	199.71
Cellulose	60.98	60.98	60.98
Lard	230.50	230.50	230.50
Mineral mix (AIN-93G)	42.68	42.68	42.68
Vitamin mix (AIN-93)	12.20	12.20	12.20
L-cystein	2.20	2.20	2.20
Choline bitartrate	3.05	3.05	3.05
GJ	-	1.0	2.0
Total	1000	1000	1000
Energy density (kcal/g)	4.6	4.6	4.6
Carbohydrates % (kcal)	41.5	41.5	41.5
Protein % (kcal)	13.5	13.5	13.5
Fat % (kcal)	45.0	45.0	45.0

GJ, green tea and java pepper mixture; HF, 45% high-fat diet; GJL, HF with 0.1% GJ; and GJH, HF with 0.2% GJ.

Table S2. Primers used for RT-qPCR

Name	GeneBank No.	Primer sequence (5′-3′)
β-actin	NM_031144	F: GGCACCACACTTTCTACAAT<br>R: AGGTCTCAAACATGATCTGG
aP2	NM_053365	F: TCACCCCAGATGACAGGAAA<br>R: CATGACACATTCCACCACCA
CD36	NM_031561	F: CCTGTGAGTTGGCAAGAAGC<br>R: AATGAGCCCACAGTTCCGAT
CPT1	NM_031559	F: TCGGCAGACCTATTTTGCAC<br>R: ATTTGGCGTAGCTGTCGATG
FAS	NM_017332	F: GCAGCAGCATGATGTAGCAC<br>R: AGTTGCACACCACAAGGTCA
PPARα	NM_013196	F: TACCTGTGAACACGATCTGA<br>R: GCTAGTCTTTCCTGCGAGTA
SCD1	NM_139192	F: GTGGCAGGGCAGGAAATAGT<br>R: CAACACCACAAGAAGCCACG
SREBP-1c	AF286470	F: AGGAGGCCATCTTGTTGCTT<br>R: GTTTTGACCCTTAGGGCAGC
UCP2	NM_019354	F: ACTGTCGAAGCCTACAAGAC<br>R: CACCAGCTCAGTACAGTTGA

aP2, adipocyte protein 2; CD36, cluster of differentiation 36; CPT1, carnitine / palmitoyl-transferase 1; FAS, fatty acid synthase; PPARα, Peroxisome proliferator-activated receptor alpha; SCD1, stearoyl-CoA desaturase 1; SREBP1-c, sterol regulatory element binding protein 1-c; UCP2, uncoupling protein 2.
